# Dual-Arch Prosthesis: Revolutionizing Partial Dentate Rehabilitation

**DOI:** 10.7759/cureus.62051

**Published:** 2024-06-10

**Authors:** Ekta M Kanojia, Anjali Bhoyar, Surekha A Dubey, Sheetal R Khubchandani, Mahima S Agrawal, Rucha Chiddarwar

**Affiliations:** 1 Prosthodontics and Crown and Bridge, Sharad Pawar Dental College and Hospital, Datta Meghe Institute of Higher Education and Research, Wardha, IND

**Keywords:** cross arch stabilization, dual-arch prosthetic solutions, masticatory performance, overdentures, cpd - kennedy class i mod 1

## Abstract

The utilization of natural teeth as denture abutments offers a significant advantage in retarding the residual ridge resorption (RRR). This approach distributes stress concentration between the denture-bearing areas and abutment teeth, thereby mitigating issues such as loss of sensory feedback, compromised mastication, and aesthetic concerns. Overdentures, by providing additional support and stability, play a pivotal role in reducing RRR while enhancing stability and retention. A cast partial denture (CPD) becomes the first choice in cases of long edentulous span where cross-arch stabilization is required. The simplicity of insertion, removal, and maintenance, coupled with effective oral hygiene practices, make CPDs a practical solution. This case presentation illustrates the successful prosthetic rehabilitation of a partially dentate patient through the implementation of a maxillary overdenture and mandibular CPD underscoring the efficacy of this treatment modality in achieving optimal outcomes. The combination of these prostheses restored the masticatory function, improved the aesthetics, and enhanced the quality of life of the patient. This case highlights the effectiveness of dual-arch prosthetic solutions in achieving comprehensive rehabilitation in partially dentate patients.

## Introduction

A condition called edentulousness is characterized by the loss of a tooth, leading to adverse biomechanical and aesthetic consequences. While there has been a decline in the prevalence of complete denture (CD) wearers, the number of partially edentulous patients has risen, likely influenced by global aging trends and oral health-focused preventive measures. Prosthetic solutions provide a range of choices for restoring missing tooth in partially edentulous individuals, encompassing removable partial dentures (RPDs), which can be supported by natural teeth or dental implants [1].

Achieving aesthetically and functionally successful prosthetic rehabilitation necessitates meticulous treatment planning and attention to detail. The Kennedy classification is a system used in dentistry to categorize partially edentulous arches based on the location and extent of missing teeth, aiding in the design of RPDs. It includes four main classes: class I (bilateral edentulous areas posterior to the remaining natural teeth), class II (a unilateral edentulous area posterior to the remaining natural teeth), class III (a unilateral edentulous area with natural teeth remaining both anterior and posterior to it), and class IV (a single, bilateral edentulous area located anterior to the remaining natural teeth). This classification helps in planning and communication among dental professionals [2]. Rehabilitation of partially edentulous arches presents challenges, particularly in distal extension situations classified under Kennedy's class I and class II categories [3]. In such cases, the fabrication of a fixed partial denture (FPD) is not viable due to the absence of distal abutments. While implant-supported prostheses may be considered, limitations such as inadequate bone volume and economic constraints may render them impractical. Therefore, in such scenarios, acrylic partial dentures or cast partial dentures (CPDs) are often preferred alternatives for achieving satisfactory outcomes [4].

The number and position of missing teeth significantly influence the effectiveness of prosthetic restorations in replicating natural dentition and maintaining oral functions. CPDs have demonstrated superior outcomes when compared to removable acrylic partial dentures, for example, the retention and stability of the denture, comfort of the patient, chewing efficiency, and periodontal condition of abutment teeth [5]. Conversely, conventional CD therapy may lead to suboptimal retention, stability, and patient satisfaction, thereby compromising confidence and comfort. While RPDs like CPDs are gradually becoming less favored as fixed prosthesis options become more accessible, the treatment of choice, particularly for medically compromised patients, is CPD over FPD as illustrated in the present case [6].

Overdentures offer a promising solution to address the shortcomings associated with conventional CD. They effectively mitigate issues such as denture loosening, diminished proprioception, and residual ridge resorption, thereby serving as a preventive measure against complete edentulism [7]. Overdentures prioritize the preservation of natural teeth, aligning with patient preferences and helping to maintain oral function and aesthetics. Despite advancements in dental implants, the conservative approach of preserving roots followed by overdenture placement remains relevant. The selection of treatment modalities is influenced by factors such as the condition of surrounding teeth and tissues, patient preferences, and financial considerations [8]. This case report highlights the successful rehabilitation of a partially dentate patient utilizing a maxillary overdenture and mandibular CPD, emphasizing the effectiveness of this treatment approach in attaining favorable outcomes.

## Case presentation

A female patient aged 72 years old presented to the Department of Prosthodontics and Crown and Bridge with a major complaint of edentulism and masticatory dysfunction. She reported undergoing dental extractions for six months. The areas were maxillary (left and right) posterior-most regions. The patient's reluctance to pursue invasive interventions necessitated the formulation of a conservative treatment approach. Given her edentulous state and functional impairment, the treatment plan focused on prosthetic rehabilitation to restore oral function and aesthetics. This involved the fabrication of RPDs for the missing posterior teeth, aiming to optimize masticatory efficiency and improve the patient's overall quality of life.

**Figure 1:**
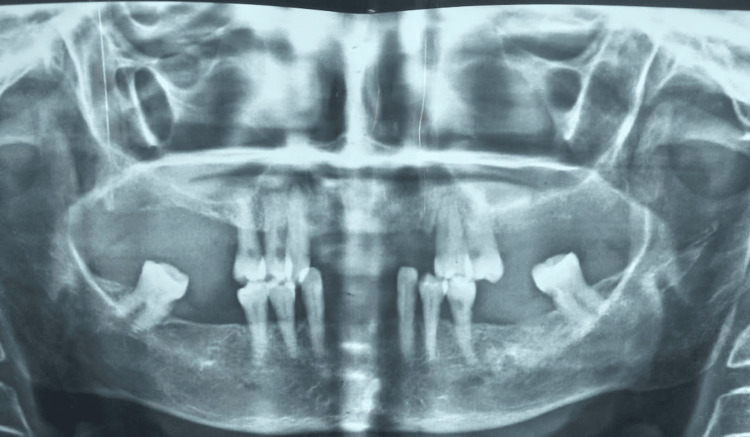
Orthopantomograph image.

Clinical examination revealed that the patient has multiple missing teeth in different locations of the oral cavity. In the maxillary arch, the first, second, and third molars were absent bilaterally, along with teeth 11, 12, 21, 22, and 23. Similarly, in the mandibular arch, teeth 31, 32, 36, 37, 41, 42, 46, and 47 were missing. Additionally, tooth 38 exhibited root caries and severe mobility, while tooth 48 remained present. The patient demonstrated adequate oral hygiene. Clinical examination findings were consistent with those from the radiographic examination, particularly evident in the orthopantomography, which indicated a poor prognosis for teeth 38 and 48. Consequently, extractions were planned (Figure 1).

**Figure 2:**
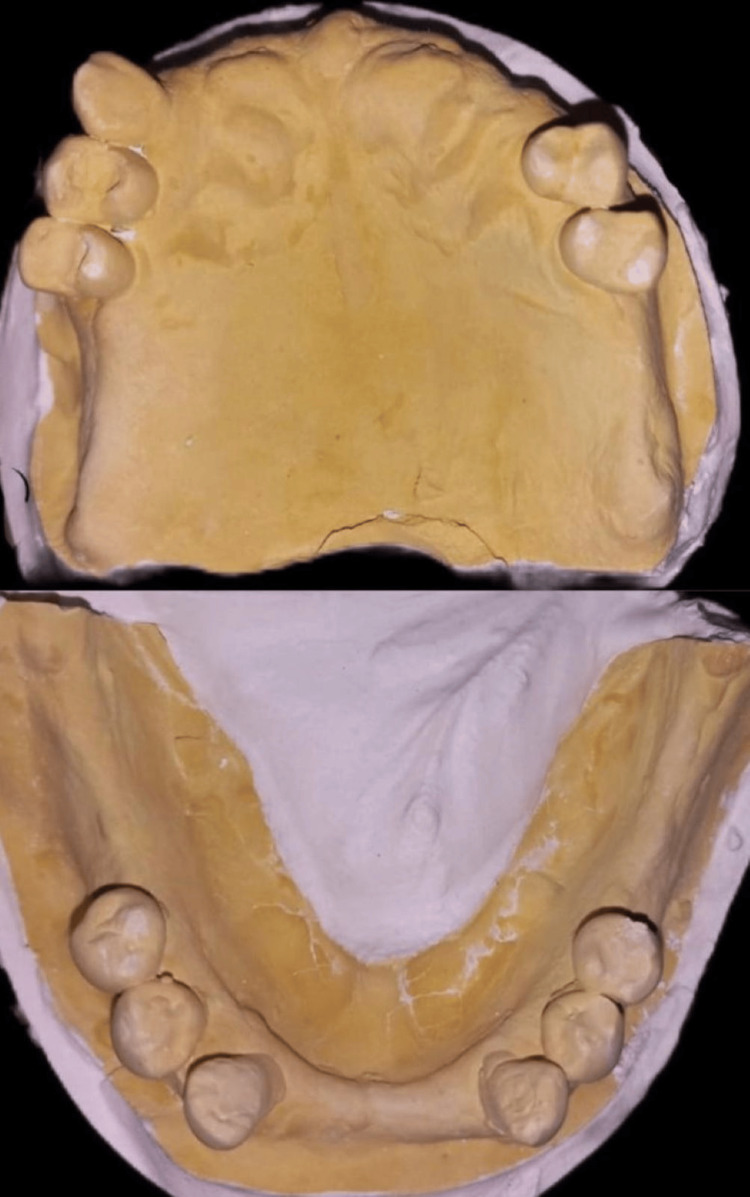
Maxillary and mandibular diagnostic cast.

Mouth preparation was done. Impressions were made in irreversible hydrocolloid material. Subsequently, dental stone diagnostic models were made (Figure 2).

**Figure 3:**
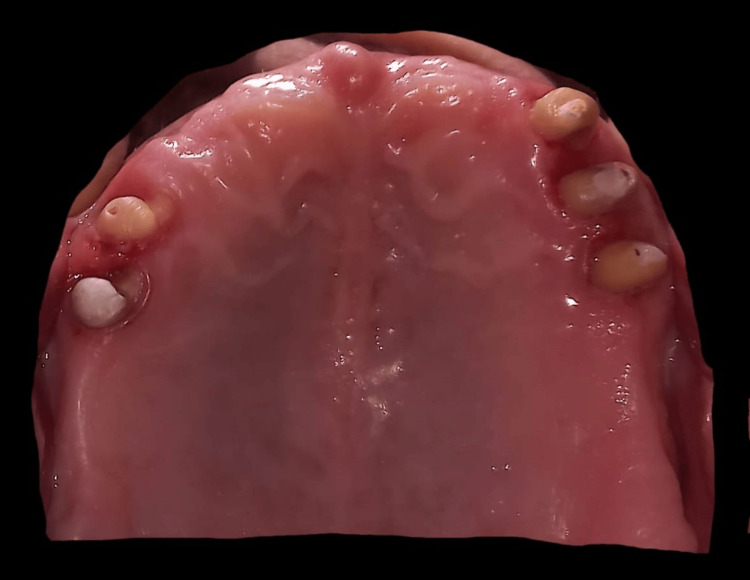
Tooth preparation done for the metal coping for tooth-supported maxillary overdenture.

The existing occlusion of the patient was recorded using bite registration. The stone models were mounted on an articulator (mean value). The models were meticulously examined for diagnostic purposes, allowing for a comprehensive assessment of the patient's dental condition and aiding in the formulation of an appropriate treatment plan. Rehabilitation was done with a tooth-supported overdenture for the maxillary arch. The mandibular arch was rehabilitated with a CPD - Kennedy Class I Mod 1. Endodontic (root canal) therapy was deemed necessary for all maxillary and mandibular teeth due to proximal caries, periapical infection, and chronic pain experienced by the patient. In the maxillary arch, teeth were prepared in a dome-shaped manner with an approximate height of 3-4 mm, using tapered, round-end diamond points. Chamfer finish lines were incorporated subgingivally (Figure 3) to ensure optimal fit and restoration longevity.

**Figure 4:**
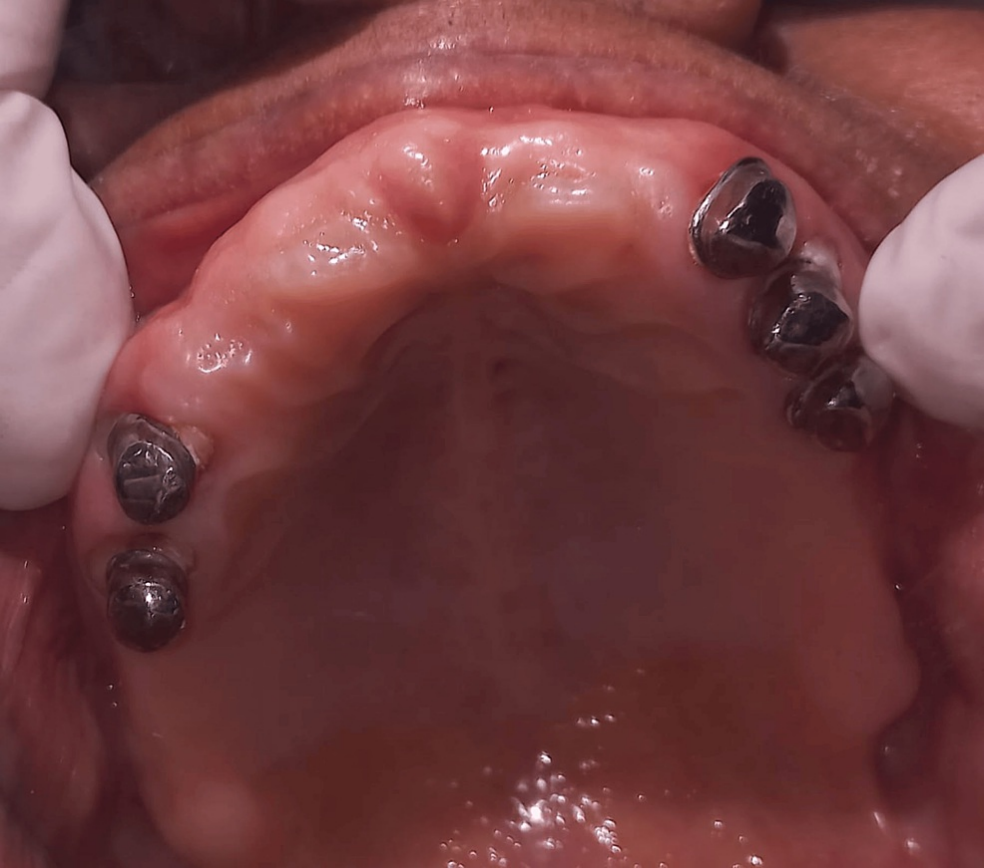
Metal coping cemented on the maxillary cast for overdenture.

Following gingival retraction in the maxillary arch, a final impression was obtained using additional silicone material employing the two-step impression technique. This impression served as the basis for creating the master model, which in turn facilitated the fabrication of copings for subsequent restorative procedures (Figure 4).

**Figure 5:**
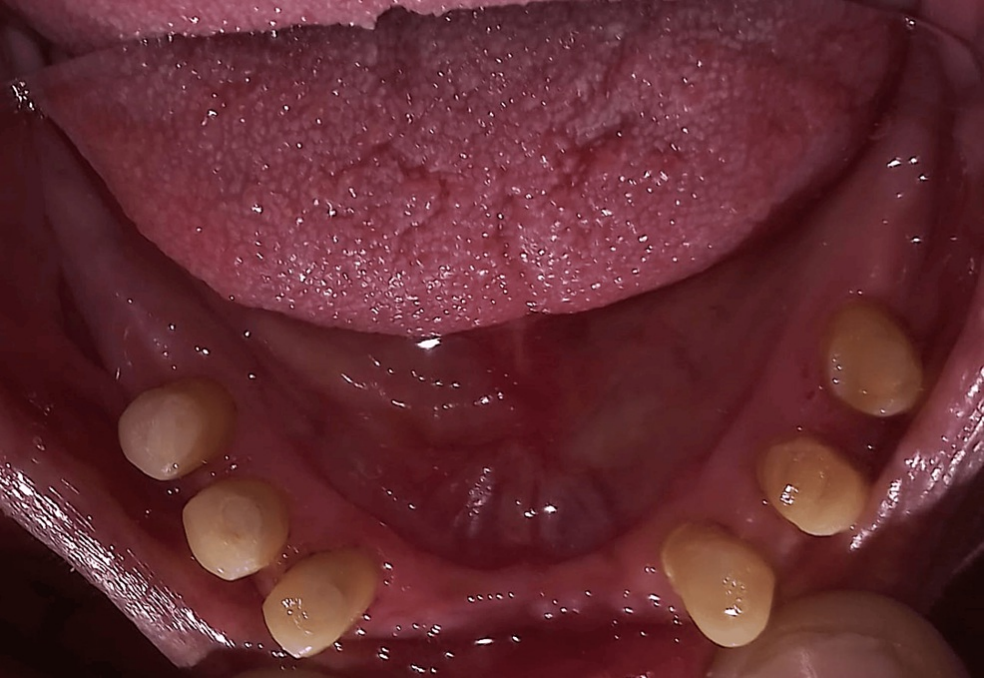
Tooth preparation done for the PFM crown with teeth 33, 34, 35, 43, 44, and 45. PFM: porcelain-fused-to-metal

In the mandibular arch, all teeth were prepared for porcelain-fused-to-metal (PFM) crowns, with teeth 33, 34, 35, 43, 44, and 45 designated for restoration (Figure 5).

**Figure 6:**
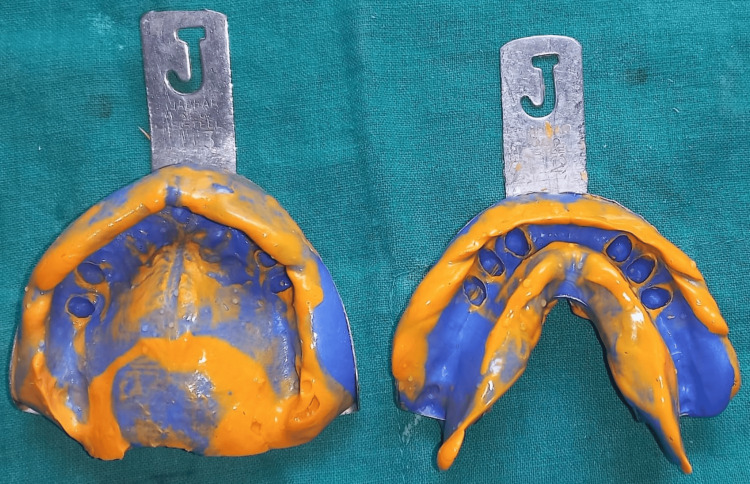
Final impression made for the master cast.

Rest seats were incorporated onto the wax pattern of teeth 33, 35, 43, and 45, with guiding planes prepared accordingly. Subsequently, the crowns were cemented in place. Following crown cementation, the final impression was made (Figure 6).

**Figure 7:**
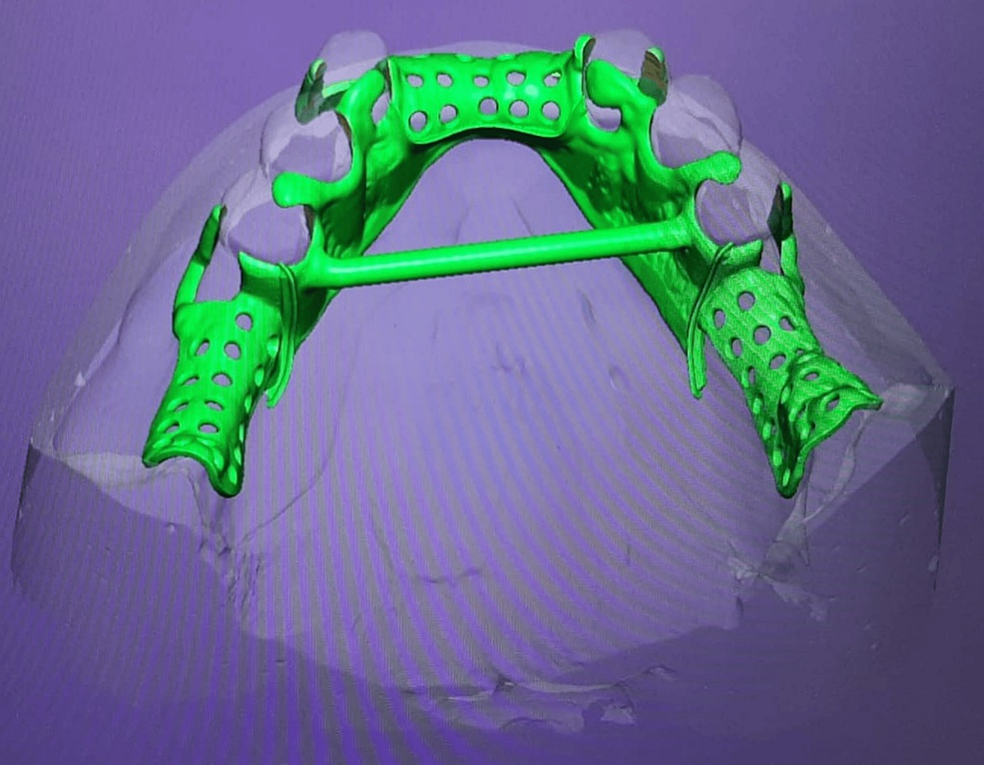
Using exocad digital software for designing of the mandibular CPD. CPD: cast partial denture

The resulting secondary cast was surveyed to examine and design the cast partial framework. Digital scans were used to finalize the design of the CPD (Figure 7).

**Figure 8:**
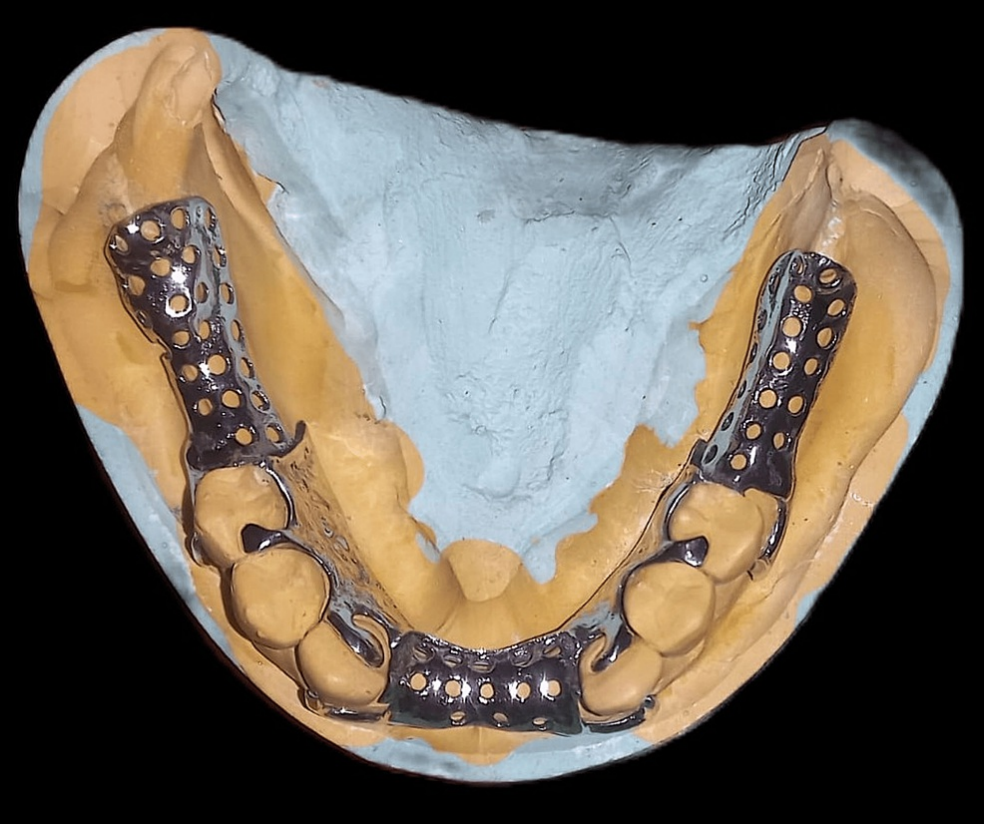
Try-in of the metal framework on the cast.

**Figure 9:**
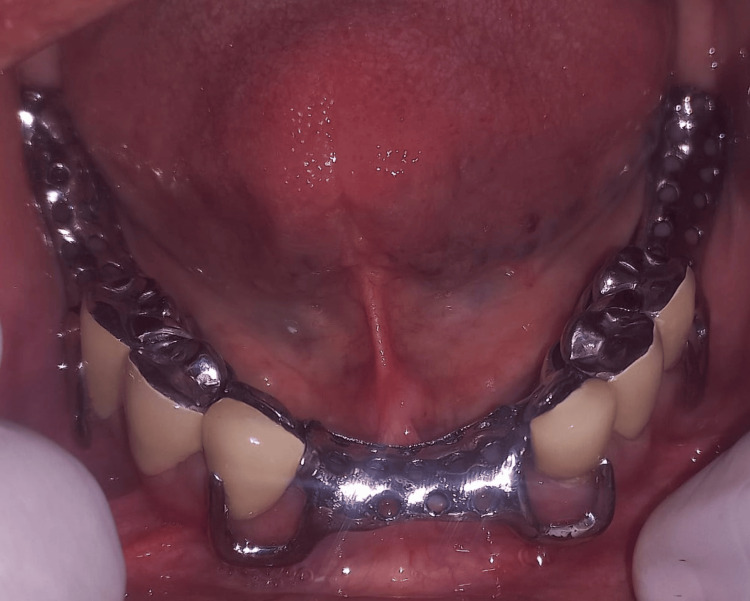
Try-in of metal framework intraorally.

Addition silicone materials of two consistencies, namely, heavy and light, were used for making the final impression. Then, a master cast was poured. Duplication of the cast was done using a reversible hydrocolloid to obtain a refractory cast made up of investment material. The design of the CPD was formulated, and it was transferred to the refractory cast after which a wax pattern was fabricated. This wax framework was subjected to casting, and Co-Cr alloy was employed. The metal framework after casting was finished and polished (Figure 8 and Figure 9). Framework was then assessed intraorally.

**Figure 10:**
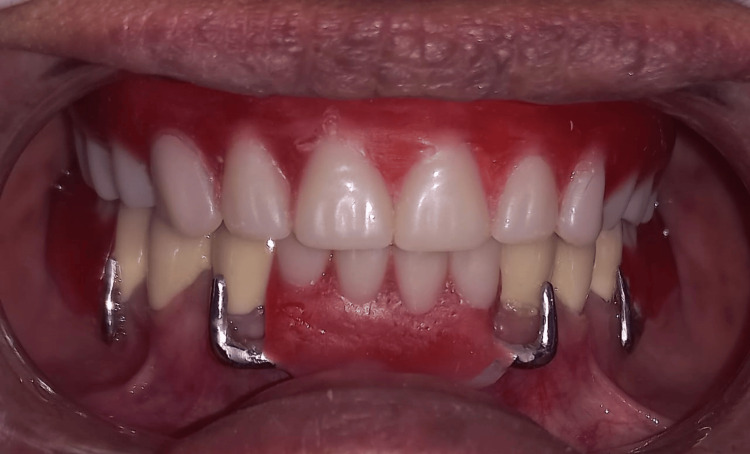
Try-in procedure image.

Jaw relation was recorded using occlusal rims. Mounting of the recorded relation was done using an articulator (semi-adjustable). Try-in was done to ensure proper fit and function (Figure 10).

**Figure 11:**
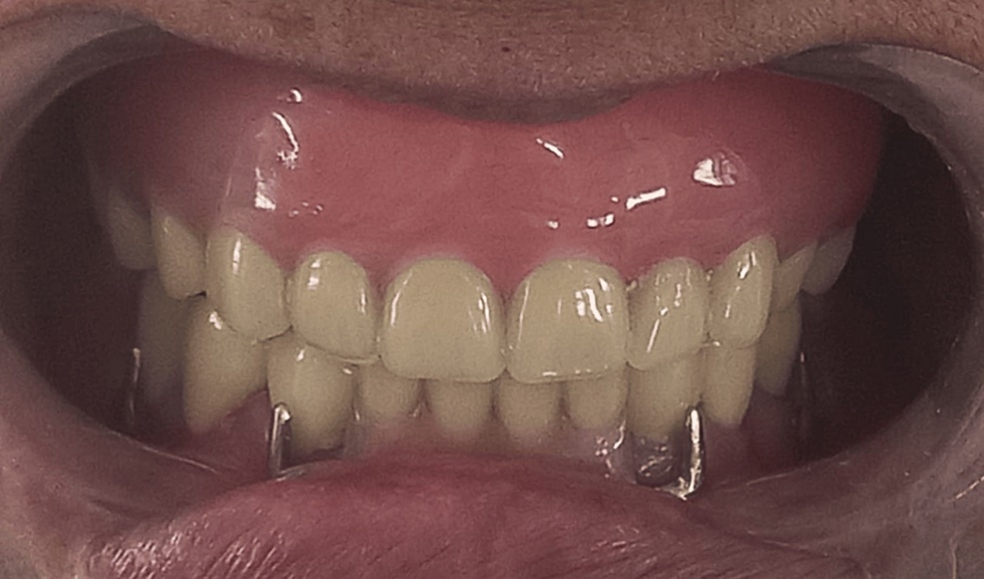
Final denture insertion procedure.

Finally, Figure 11 displays the prostheses intraorally, showcasing the completed dental restoration.

**Figure 12:**
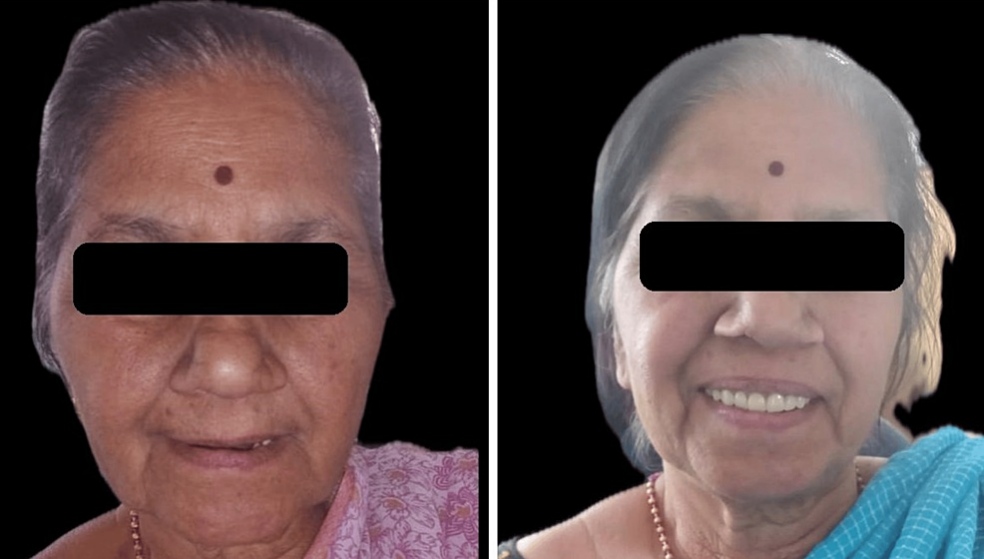
Preoperative and postoperative photograph.

Figure 12 shows the preoperative and postoperative images of the patient.

## Discussion

For this patient, a maxillary overdenture and a mandibular CPD were selected as treatment modalities owing to their superior stability and retention properties, facilitating optimal distribution of stress and proprioceptive feedback through extant natural dentition. Conversely, alternative therapeutic approaches encompassed the removal of teeth which are present and then a conventional complete set of denture placement [9]. However, such an approach was eschewed due to its potential to diminish proprioceptive input and compromise the structural support conferred by the remaining natural dentition and associated periodontal ligaments. The full-mouth implant-supported prosthesis was precluded as a treatment avenue, given the patient's medical comorbidities and aversion to surgical interventions [10].

Nevertheless, tooth-supported overdentures present certain drawbacks, including the imperative for meticulous oral hygiene to avert periodontal and caries afflictions. These overdentures typically possess larger, more contoured structures, potentially encroaching upon inter-occlusal space [11]. Furthermore, RPDs exhibit limitations, such as patient adaptation challenges and the requisite adjustment period, plaque accumulation on abutment teeth, predisposition to periodontal pathology, and carious lesions. Additionally, inadequately designed RPDs may manifest aesthetic concerns due to visible metal components. The prospect of complete tooth loss is profoundly distressing for patients, precipitating a decline in morale by underscoring dependence and aging [11]. In such instances, the consistent integration of overdentures as a preventive prosthodontic intervention is advocated within dental practices due to their multifarious advantages.

A study has been conducted by Rissin et al. comparing masticatory performance among individuals with natural dentition, CD, and overdentures. Their findings revealed that masticatory efficiency was superior in patients wearing overdentures compared to those with CD [12]. Additionally, Crum and Rooney conducted a five-year study where cephalometric radiographs illustrated vertical bone loss (0.6 mm average) in the region of the anterior mandible of overdenture wearers, contrasting with a 5.2 mm loss observed in CD wearers [13]. Another study found that the existence of RPDs had a substantial impact on nutritional consumption, implying that restoring edentulism might encourage patients towards a healthy plan of diet [14]. Partial tooth loss has been shown to have a negative influence on food acceptance, similar to how edentulism impacts diet quality and nutritional status. Consequently, removable treatment modalities are favored for patients unable to undergo fixed prosthetic procedures despite limitations [15].

Moreover, retaining natural teeth preserves proprioception in overdenture wearers, contributing to enhanced satisfaction and patient acceptability. Thus, this case presentation serves to validate the partially dentate successful rehabilitation through the utilization of a maxillary overdenture and mandibular CPD.

## Conclusions

This case presentation demonstrates the successful prosthetic rehabilitation of a partially dentate patient through the implementation of a maxillary overdenture and mandibular CPD. By utilizing these treatment modalities, optimal outcomes in terms of function, aesthetics, and patient satisfaction were achieved. The use of a maxillary overdenture helped preserve natural teeth and mitigate issues associated with complete edentulism, while the mandibular CPD provided stability, retention, and improved masticatory function. Overall, this case underscores the efficacy of combining these prosthetic options in addressing the complex needs of partially dentate individuals, ultimately enhancing their quality of life and oral health.
